# A Novel msDNA (Multicopy Single-Stranded DNA) Strain Present in *Yersinia frederiksenii* ATCC 33641 Contig01029 Enteropathogenic Bacteria with the Genomic Analysis of It's Retron

**DOI:** 10.4061/2011/693769

**Published:** 2011-11-17

**Authors:** Rasel Das, Tadashi Shimamoto, Md. Arifuzzaman

**Affiliations:** ^1^Department of Biochemistry and Biotechnology, University of Science and Technology Chittagong (USTC), Foy's Lake, Chittagong 4202, Bangladesh; ^2^Laboratory of Food Microbiology and Hygiene, Graduate School of Biosphere Science, Hiroshima University, Higashi-Hiroshima, Hiroshima 739-8528, Japan

## Abstract

Retron is a retroelement that encodes msDNA (multicopy single-stranded DNA) which was significantly found mainly in Gram-negative pathogenic bacteria. We screened *Yersinia frederiksenii* ATCC 33641 contig01029 for the presence of retroelement by using bioinformatics tools and characterized a novel retron-Yf79 on the chromosome that encodes msDNA-Yf79. In this study, we perceived that, the codon usage of retron-Yf79 were noteworthy different from those of the *Y. frederiksenii* genome. It demonstrates that, the retron-Yf79 was a foreign DNA element and integrated into this organism genome during their evolution. In addition to this, we have observed a transposase gene which is located just downstream of retron-Yf79. So, the enzyme might be responsible for the transposition of this novel retron element.

## 1. Introduction

For the past 21 years, it has been shown that some pathogenic Gram-negative bacteria strains contain genetic elements called retrons. Retron is a retroelement consisting of *msr*, which encodes the RNA part of msDNA, *msd*, which encodes the DNA part of msDNA, and the *ret* gene for reverse transcriptase (RT) [1]. The reverse transcriptase (RT) was originally discovered in virus [2] as an essential enzyme for the replication of retroviruses. Since the discovery of RT in myxobacteria [3] and *Escherichia coli* [4] an intriguing question have been raised concerning its origin and function in the prokaryotes [5]. 

The msDNA (multicopy single-stranded DNA) is composed of a small, single-stranded DNA, linked to a small, single-stranded RNA molecule. The 5′ end of the DNA molecule is joined to an internal guanine base (G) residue of the RNA molecule by a unique 2′, 5′-phosphodiester bond [6]. Since msDNA was originally discovered in the Gram-negative soil bacterium, *Myxococcus xanthus* [7] it was also isolated from aggregative adherence *E. coli* (AAEC) [8], a classical enteropathogenic *E. coli* (EPEC) [9] and more recently from *Vibrio cholerae* [10], *Salmonella enterica* serovar Typhimurium [5], *V. parahaemolyticus *and *V. mimicus *(Shimamoto T, 2003, unpublished data). Hence, RT might have a role in diversification of pathogenic bacteria genomes. 

Although msDNAs have been isolated over the pathogenic Gram-negative bacteria, in this study we characterized a novel retron region by screening the complete genome sequence of *Yersinia frederiksenii* [11] which encodes *msr*, *msd* with a *ret* gene by best hits RT sequence similarity along with *V. cholerae, V. parahaemolyticus and S.* Typhimurium. These provide insight into the important roles of this mysterious element in these bacteria species.

## 2. Materials and Methods

### 2.1. Genomic Analysis of Retron-Yf79

#### 2.1.1. Sequence Retrieval

To determine the particular place of retron-Yf79, the complete nucleotide genome sequence of *Yersinia frederiksenii* ATCC 33641 contig01029 was retrieved from the national center for biotechnology information (NCBI) resource at (http://www.ncbi.nlm.nih.gov/) with the following accession number (AALE02000035) [11]. To investigate an evolutionary relationship among amino acid sequence of reverse transcriptases from *Y. frederiksenii*, *V. cholerae, V. parahaemolyticus *and *S.* Typhimurium; were collected from ExPASy proteomics server at (http://expasy.org/). In addition, the 16S ribosomal RNA (16S rRNA) nucleotide sequences of *Y. frederiksenii*, *V. cholerae, V. parahaemolyticus *and *S.* Typhimurium were collected from the kyoto encyclopedia of genes and genomes (KEGG) organism database available at GenomeNet server, Japan (http://www.genome.jp/) to observe the possible evolutionary scenario among those species.

#### 2.1.2. Sequence Alignment

The genomic organization of *msd-msr* region of retron-Yf79 was determined according to their nucleotide sequences analyzing, that is, the presence of conserved region nucleotides with other *msr-msd *coding regions which have been isolated from various pathogenic bacteria- (*V. cholerae, V. parahaemolyticus* and* S*. Typhimurium) by using (ClustalW) program available at (http://www.genome.jp/tools/clustalw/), Japan [12]. To evaluate the similarity of RT-Yf79 with others RT-Vc95 from *V. cholerae *[10], RT-Vp96 from *V. parahaemolyticus* (Shimamoto T, 2003, unpublished data) and RT-St85 from *S. *Typhimurium [5], the alignment program was utilized at the site (http://www.genome.jp/tools/clustalw/) [12], after determining the best hit of RTs sequence similarity search by the BLAST program at NCBI Blast homepage (http://blast.ncbi.nlm.nih.gov/Blast.cgi).

#### 2.1.3. Structure Prediction and Codon Bias Analysis

The DNA and RNA secondary structures of msDNA-Yf79 were predicted by using the database-(http://www.ncrna.org/centroidfold/) [13]. The promoter sequence of retron-Yf79 was predicted on the basis of the conserved promoter sequences [14]. To appraise whether the retron is a foreign DNA element, the codon bias was carried out. The codon bias of retron-Yf79 and the whole organism genome was resolute by using codon usage database-(http://www.kazusa.or.jp/codon) [15].

#### 2.1.4. Phylogenetic Analysis

To evaluate the origin and similarity of RT-Yf79 from *Y. frederiksenii*, phylogenetic tree was constructed by using other RTs from (*V. cholerae, V. parahaemolyticus *and *S. *Typhimurium). These amino acid sequences were aligned along with each other by using (ClustalW) at (http://www.genome.jp/tools/clustalw/), Japan [12]. The sequence alignment was performed under default conditions and the phylogenetic tree was constructed by the neighbor-joining method. The phylogenetic tree of 16S ribosomal RNAs was also constructed based on their nucleotide sequences by using same database available at (http://www.genome.jp/tools/clustalw/), Japan [12].

## 3. Results

### 3.1. The Structure of msDNA-Yf79

**Figure 1:**
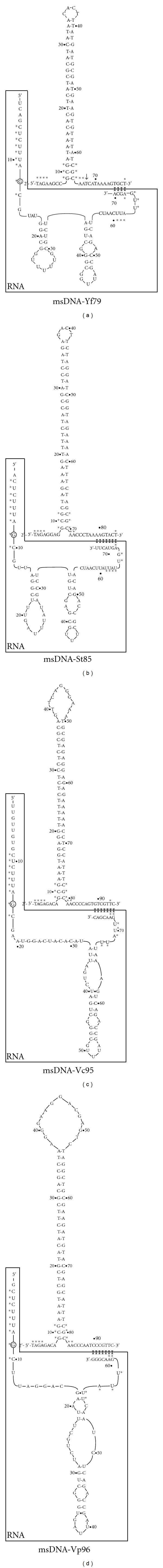
Possible secondary structures of multicopy single-stranded DNA (msDNAs) from pathogenic bacteria. (a) The branching guanine base (G) residue at position 12 in RNA portion of msDNA is circled and forming a 2′, 5′-phosphodiester bond (a). Both the DNA and RNA secondary stem loop structures were suggested on the basis of their sequences. The RNA portion was boxed and the numbers of RNA and DNA were begun from 5′ ends. The conserved nucleotides are indicated by stars in all msDNAs. (a) The msDNA-Yf79 is predicted from *Yersinia frederiksenii* [11], (b) msDNA-St85 is isolated from *S*. Typhimurium [5], (c) msDNA-Vc95 is from *V. cholerae* [10], and (d) msDNA-Vp96 is from *V. parahaemolyticus *(Shimamoto T, 2003, unpublished data). The arrow indicates thymine base (T) at position 67 in the DNA part of msDNA-Yf79 (a).

Analysis of *msd* nucleotide sequence showed that the DNA part of msDNA found in *Y. frederiksenii* is predicted to consist of 79 bases of a single-stranded DNA, and hence it was named as msDNA-Yf79, and the RNA part of msDNA-Yf79 consists of 70 bases encoded by *msr* gene of retron-Yf79 (Figure 1(a)). Furthermore, the guanine base (G) residue at position 12 of the RNA molecule branched out by a unique 2′, 5′-phosphodiester link (Figure 1(a)). The msDNAs isolated from other bacteria contains at least one mismatched base pair in their DNA stems which could be mutagenic [16, 17]. However, in this study we observed that the DNA structure of msDNA-Yf79 contains no any mismatched base pair as like as most of msDNAs were isolated from other pathogenic bacteria (Figure 1(a)). Further, the msDNA-Yf79 shared a number of conserved nucleotide sequences with other msDNAs (msDNA-St85,-Vc95 and -Vp96) (Figure 1), except thymine (T) at position 67 in DNA part of msDNA-Yf79 (Figure 1(a)). 

### 3.2. Genomic Organization of Retron-Yf79

**Figure 2:**
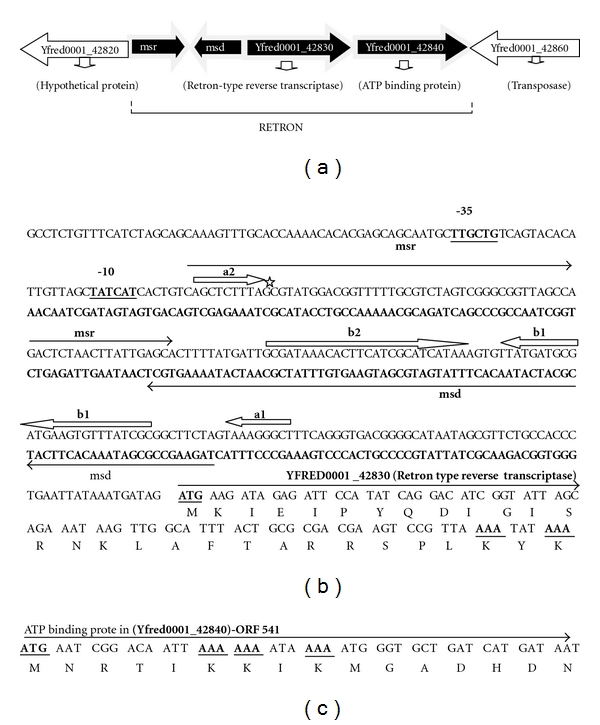
Genomic organization of retron-Yf79 in whole genome of *Yersinia frederiksenii* chromosome (a) and the *msr-msd* nucleotide sequence along with RT gene in (b): the −35 and −10 conserved promoter sequences are underlined and located at just upstream of *msr-msd* coding sequence. Inverted repeats, a1/a2 and b1/b2, are indicated by arrows, while the conserved guanine (the branching G) at the 12th position of the *msr* is shown by star on top of the G. The partial N-terminal amino acid sequences of both RT and ORF-541 are indicated, and the ATG (methionine) and AAA (lysine) are bold and underlined in (b and c).

The retron-Yf79 consists of nucleotide sequence of about 2.8 Kb, and the retron element is transcribed from the −35 and −10 conserved promoter sequence located 5 bp upstream to the *msr-msd* coding region (Figures 2(a) and 2(b)). In addition, two open reading frames (ORFs) were located just downstream of *msr* and *msd* coding sequence, one is RT-Yf79 encoded retron-type reverse transcriptase having 310 amino acids, and another one is ORF-541 which encoded a putative ATP binding protein containing 541 amino acids (Figure 2(a)). The upstream and downstream regions of retron element also contained yfred0001_42820 gene that encoded a hypothetical protein (356 AAs) and Yred0001_42860 gene that encoded a transposase (308 AAs), respectively (Figure 2(a)).

### 3.3. Codon Usage of Retron-Yf79

To identify the origin of RT-Yf79 and ORF-541 genes in *Y. frederiksenii* genome, the codon usages were carried out. It revealed that the RT-Yf79 and ORF-541 genes used AAA codon for lysine with a frequency of 55% and 74%, respectively, but the *Y. frederiksenii* genome only used AAA codon for lysine with a frequency of 20% of the time (data not shown). Present observation suggested that the retron-Yf79 is a foreign DNA element and probably acquired in this organism chromosome from other ancestral species during their evolution times.

### 3.4. Comparative Study of RT-Yf79 with Other ret Genes from Different Pathogenic Bacteria

**Figure 3:**
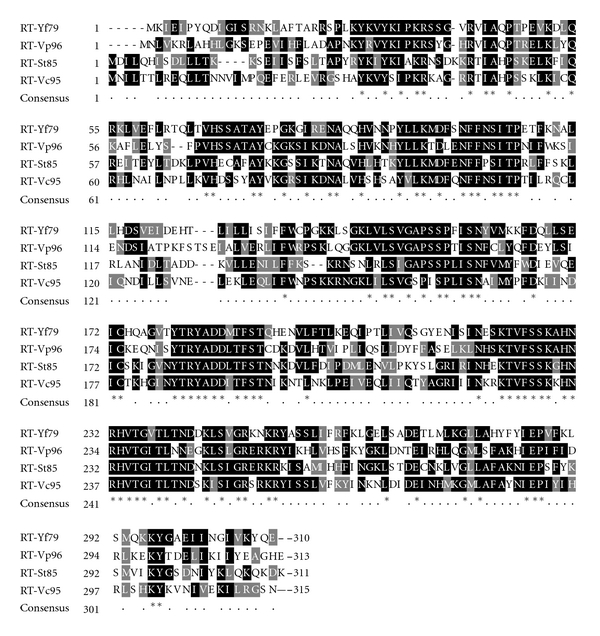
Comparison of the amino acids sequence alignment of the RT-Yf79 with the three highest identity RT sequences: RT-Vc95 (44% identity), RT-Vp96 (45% identity), and RT-St85 (43% identity). Amino acids conserved in all four RTs are marked with asterisks and black colors. Conserved and well-conserved amino acids residues are marked with dots and the number of amino acids of each RT was written at the end of the alignment.

The RT-Yf79 encoded by the retron-Yf79 consists of 310 AA residues. Surprisingly, all retron RTs in pathogenic bacteria were shown to have the highest identities to RT-Yf79: RT-Vc95 (from *V. cholerae,* 44% identity), RT-Vp96 (from *V. parahaemolyticus,* 45% identity), and RT-St85 (from *S*. Typhimurium, 43% identity) when these RTs were compared with each other by using multiple amino acids alignment (Figure 3). These four RTs shared approximately similar number of amino acids (Figure 3). In addition, they all shared a conserved domain along with each other (data not shown).

### 3.5. Phylogenetic Analysis of RTs and 16S Ribosomal RNA Gene Sequences

**Figure 4:**
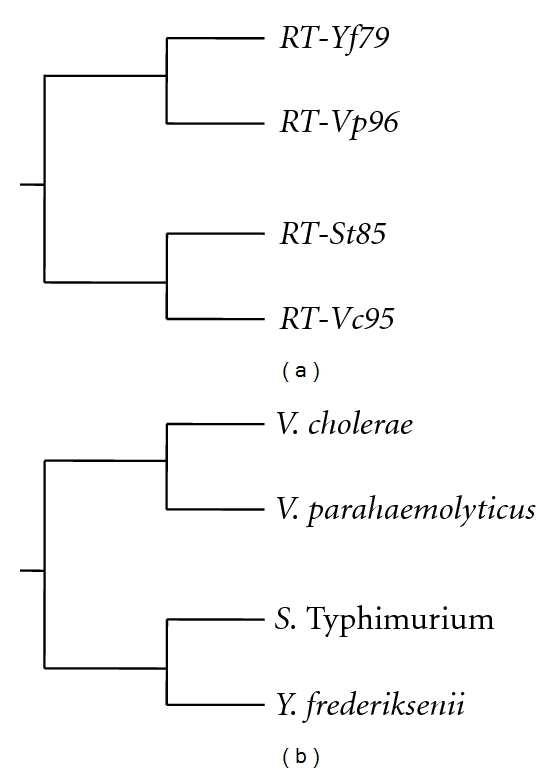
Phylogenetic trees among *Y. frederiksenii, V. cholerae, V. parahaemolyticus, and S*. Typhimurium based on the RT (a) and the 16S ribosomal RNA genes (b). The trees were constructed by using the neighbor-joining (NJ) method in the CluslalW program. The following ExPASy accession numbers for the RT sequences were used in the phylogenetic construction: *Y. frederiksenii* RT-Yf79-C4SUU2, *V. cholerae* RT-Vc95-Q9S1F2, *V. parahaemolyticus-*Q8L0W6, and *S*. Typhimurium- E7UVY4. The following GenomeNet accession numbers for the 16S rRNA sequences were used in the phylogenetic construction: *Y. frederiksenii-*NR_027544.1, *V. cholerae*-2614447, *V. parahaemolyticus-*1187490 and *S*. Typhimurium-1251767.

To observe the genomic diversity of *ret* genes and orthologous 16S ribosomal RNA genes (from *Y. frederiksenii*, *V. cholerae, V. parahaemolyticus *and *S*. Typhimurium) phylogenetic trees were constructed by using ClustalW at (http://www.genome.jp/tools/clustalw/), Japan [12] (Figure 4). The phylogenetic tree analysis showed a fundamental diversity among the *ret* genes in relation to the host bacteria (*Y. frederiksenii*) species as RT-Yf79 from *Y. frederiksenii* [11] was closely related to RT-Vp96 from *V. parahaemolyticus* (Shimamoto T, 2003, unpublished data) rather than to the RT-St85 from *S*. Typhimurium [5] and RT-Vc95 from *V. cholerae* [10] of pathogenic bacteria as RT-St85 was closely related to the RT-Vc95 (Figure 4(a)). Although both RT-Vc95 and RT-Vp96 were from *Vibrio* species, both were diverged from each other as they were closely related to RT-St85 and RT-Yf79, respectively (Figure 4(a)). The 16S ribosomal RNA phylogenetic analysis suggested that, these pathogenic bacteria genomes might acquire these retron elements during their evolution (Figure 4(b)).

## 4. Discussion

In this study, we demonstrated that a new msDNA-Yf79 exists in *Y. frederiksenii* ATCC 33641 contig01029 cell types and compared it's properties to that of St85 [5], Vc95 [10] and Vp96 (Shimamoto T, 2003, unpublished data). The retron-Yf79 was responsible for the production of msDNA-Yf79 in *Y. frederiksenii* Gram-negative pathogenic bacteria strain.

However, the gene organization of retron-Yf79 was similar to those found in *E. coli *(retron-Ec83 and -Ec78) [8, 9], that is, contained only two open reading frames (ORFs) in their retroelement. On the other hand, the gene organization of retron-Vc95 [10] and retron-Vp96 (Shimamoto T, 2003, unpublished data) were completely different as they contained a third ORFs. The msDNA-Yf79 has a sequence similarity to msDNA-St85, msDNA-Vc95 and msDNA-Vp96 as these msDNAs shared a number of highly conserved bases in their nucleotide sequences, indicating that they might be descended from a common origin (i.e., from a common ancestor). The presence of the conserved guanine base (G) at position 12 in RNA part of msDNA-Yf79 which involved in branch formation via a 2′, 5′-phosphodiater link in DNA-RNA complex (Figure 1(a)). Lima and Lim suggested that the fact that the mutation in guanine base (G) prevents msDNA synthesis and the primary product of reverse transcription may be a branched DNA-RNA compounds [9], which supports our observation. 

Furthermore, it was quite interesting that stem structure of msDNA-Yf79 did not contained any mismatched base pair like most of the msDNA isolated from other pathogenic bacteria. Moreover, the codon usage of this retron element and also the phylogenetic analysis of RTs and 16S rRNA from pathogenic bacteria revealed that this retron was a foreign DNA element. The downstream of retron element-Yf79 contained a transposase gene indicating that this enzyme might be participated in transposition of this novel retron element in the genome. 

We resolved after consideration to look closely the nucleotide sequence of this retron-Yf79 in *Y. frederiksenii* because this organism has generated significant value in the role of pathogenicity. Functions of msDNA are still not clear. However, this DNA-RNA complex which was identified in Gram-negative pathogenic bacteria may support its role in the process of pathogenicity. In addition, retron element may play an essential role for adaptation of such bacteria in different stressful conditions by changing the expression of their regulatory social behavior under which conditions that expression is densely populated. Further experiment will be required for demonstrating the functions of msDNA, which may be opened a new arena in the process of pathogenicity or adaptation in stressful conditions.
